# Fe_3_O_4_/Diatomite-Decorated Cotton Evaporator for Continuous Solar Steam Generation and Water Treatment

**DOI:** 10.3390/ma15176110

**Published:** 2022-09-02

**Authors:** Zhi Bai, Haifeng Xu, Bo Yang, Jixin Yao, Guang Li, Kai Guo, Nan Wang, Nannan Liang

**Affiliations:** 1 School of Mechanical and Electronic Engineering, Suzhou University, Suzhou 234000, China; 2 School of Information Engineering, Suzhou University, Suzhou 234000, China; 3 School of Physics and Electronic Information, Huaibei Normal University, Huaibei 235000, China; 4 Universities Joint Key Laboratory of Photoelectric Detection Science and Technology in Anhui Province, Hefei Normal University, Hefei 230601, China; 5 Anhui Province Key Laboratory of Simulation and Design for Electronic Information System, Hefei Normal University, Hefei 230601, China; 6 Anhui Key Laboratory of Information Materials and Devices, Institute of Physical Science and Information Technology, School of Materials Science and Engineering, Anhui University, Hefei 230601, China; 7 Key Laboratory of Structure and Functional Regulation of Hybrid Materials of Ministry of Education, Institute of Physical Science and Information Technology, School of Materials Science and Engineering, Anhui University, Hefei 230601, China; 8 Anhui Provincial Engineering Laboratory on Information Fusion and Control of Intelligent Robot, Wuhu 241002, China

**Keywords:** solar steam generation, Fe_3_O_4_, cotton, diatomite, desalination

## Abstract

Improving the evaporation rate of solar steam generation (SSG) has always been a research hotspot to solve the shortage of water resources. Using cotton, Fe_3_O_4_, polyvinyl alcohol (PVA) and diatomite (DM) as raw materials, DM/PVA/Fe_3_O_4_@cotton composites with both firmness and hydrophilicity were prepared. Fe_3_O_4_ has a wide range of light absorption characteristics and good photothermal conversion performance, and is an ideal photothermal conversion material. PVA enhances the adhesion between Fe_3_O_4_, cotton and DM and enhances the hardness of the sample and the internal porous structure. The existence of DM greatly improves the hydrophilicity of the sample, ensuring that the water in the lower layer can be continuously transported to the surface of the sample, and DM makes the surface of the sample rough, which reduces the reflection of sunlight and improves the efficiency of light heat conversion. Under one-sun irradiation, the temperature of the sample surface increases by 52.6 °C, the evaporation rate can reach 1.32 kg m^−2^ h^−1^ and the evaporation efficiency is 82.9%. Using this sample as the photothermal conversion layer of the SSG device, the removal rate of salt ions in seawater is more than 98% and the removal rate of heavy metal ions in sewage is close to 100%. This work provides a new idea and design method for SSG in the field of seawater desalination and sewage treatment.

## 1. Introduction

The shortage of water resources has always been one of the problems that need to be solved urgently by mankind. Facing the global shortage of water resources, scholars have invested in research to obtain purified water, and sewage purification and seawater desalination are the main methods to solve the shortage of water resources [1,2,3,4]. However, both sewage purification and seawater desalination require a large amount of energy, so clean energy and a reliable water purification method are urgently needed. Solar energy is recognized as a clean and sustainable energy source, and solar steam generation is a reliable and convenient way of water purification [5]. Generally, the solar steam generation method using solar energy has only about approximately 35% light–heat conversion efficiency, and the evaporation efficiency is low; in addition, problems such as insufficient water supply and heat loss will occur in the evaporation process [6]. In view of this, a large number of methods to optimize SSG have emerged in recent years [7]. For example, Xiao et al. cleverly designed an evaporator, including drilling holes and cutting water channels, which can accelerate the transportation and convection of water and improve evaporation due to the precipitation of salt [8]. Li et al. designed a device that combines a three-dimensional vertebral absorber and a one-dimensional waterway to improve the evaporation area and evaporation rate [9]. Generally, three-dimensional water channels often produce heat conduction with water bodies; therefore, using two-dimensional water channels based on fiber paper and one-dimensional water channels based on cotton can reduce heat loss [10,11,12,13]. To further reduce heat loss, a layer of insulating material, such as polyethylene foam, is placed between the photothermal conversion layer and the water to prevent the material from contacting the water, thereby preventing heat loss [14,15,16,17,18,19,20].

Cotton has strong hydrophilicity, low thermal conductivity and good stability, and is considered to be a good precursor of SSG [21]. Due to the effect of the capillary phenomenon, cotton can transport a continuous flow of water to the thermal interface, and can also prevent the transfer of heat to water [22]. In addition to cotton, the efficiency of SSG can also be improved by selecting different photothermal conversion materials. Generally, photothermal conversion materials can be divided into plasma metals, carbon-based materials, narrow-bandgap semiconductors and their hybrids; in narrow-bandgap semiconductors such as Ti_2_O_3_, Fe_3_O_4_ and MoS_2_ [23], they have good photothermal conversion performance, and the electrons of semiconductor materials can receive photon energy to transition from the valence band to conduction band; through nonradiative relaxation, light energy is converted into heat energy. Some studies have proven that narrow-bandgap semiconductors have stronger electron-absorption ability than wide-bandgap semiconductors. Fe_3_O_4_ is the most representative narrow-bandgap semiconductor, which has a strong photothermal conversion ability in the visible light band, and has the advantages of simple preparation, low cost and low toxicity, and can be applied on a large scale [24]. Xu et al. used Ti_3_C_2_/MoS_2_ nanocomposite as the light absorption layer of SSG equipment; with the excellent light absorption rate of MoS_2_, it has excellent evaporation performance and stability [25].

SSG is a low-cost and reliable water treatment method, so many scholars have applied it to the field of water treatment. Although many materials have achieved ultrahigh light–heat conversion efficiency, salt deposition on the surface of materials blocks the water supply path and water evaporation path, which reduces the efficiency of seawater desalination to a certain extent; the traditional methods to solve the blockage are reverse osmosis and physical flushing. To reduce the impact of salt deposition, many scholars have developed new devices and materials, Song et al. mixed PVA and Fe_3_O_4_ on wood; after delignification, the wood had more hydrophilic groups, and by designing a double-layer structure with different hydrophilicity, it had stronger salt-resistance stability [26]. Chen et al. used Fe_3_O_4_ load to form a film on the core shell; this film was directly on the air–water surface without using other supporting materials, and the film could be recycled by using an external magnetic field, reducing pollution [27].

Here, we report an SSG system based on diatomite/PVA/Fe_3_O_4_@cotton, which is used for seawater desalination and sewage treatment. The samples made of diatomite, PVA, Fe_3_O_4_ and cotton are used as the system photothermal conversion layer; the photothermal material layer is placed on the polyethylene foam on the surface of seawater; and a cotton wick is used between the photothermal conversion layer and seawater to ensure that seawater is sent to the material surface through the capillary phenomenon to complete evaporation. Mixing PVA between cotton and Fe_3_O_4_ not only increases the adhesion between them and the mechanical hardness of the material but also increases the porosity and specific surface area; diatomite is used to improve the hydrophilicity of the evaporator. In this work, using diatomite/PVA/Fe_3_O_4_@cotton with a radius of 2 cm and using seawater with a mass fraction of 3.5 wt% as the water source to be purified, the evaporation rate under one-sun irradiation is 1.32 kg m^−2^ h^−1^, and an evaporation efficiency of 82.9% is achieved. Many experiments using this sample have found that its evaporation performance is stable, the designed system has a simple preparation method and low cost and is suitable for the practical application of large-scale SSG, and it has great market prospects in the fields of sewage purification and seawater desalination.

## 2. Experimental Section

### 2.1. Materials

Cotton was purchased from China Anqing Jinghuang Nursing Supplies Co., Ltd.(Anqing, China), polyvinyl alcohol (PVA) and diatomite were obtained from Sinopharm Chemical Reagent Co., Ltd. (Shanghai, China), and Fe_3_O_4_ was purchased from China Qinghe Xingsheng Metal Materials Co., Ltd. (Jinan, China), All chemicals were used as received without further purification.

### 2.2. Preparation of Samples

**Figure 1:**
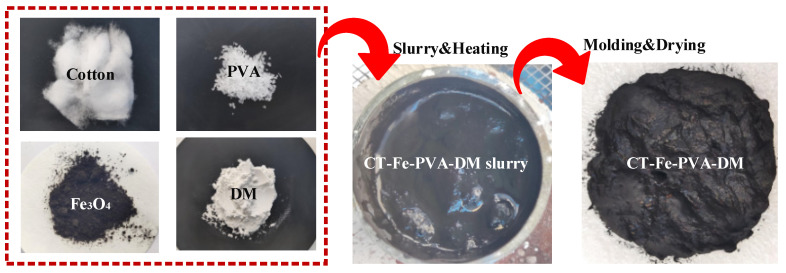
Preparation process of CT-Fe-PVA-DM.

Tear the cotton into cotton wadding and soak it in water according to the weight ratio of cotton: Fe_3_O_4_ of 1.5:1; after stirring for 6 h, add diatomite of the same quality as cotton and 2 g PVA to the above mixed solution, heat it to 80 °C and keep stirring until the water is about to evaporate dry. Then, put the above material into the mold and compress it into shape. Finally, put it into an oven at 80 °C and dry it for 12 h, take it out and cool it to room temperature for testing, and record it as CT-Fe-PVA-DM; the preparation process is shown in Figure 1. For comparison, the samples made without diatomite in the same way are recorded as CT-Fe-PVA, the samples made without PVA and diatomite are recorded as CT-Fe, and the samples made only with cotton are recorded as CT.

### 2.3. Characterization of Samples

SEM (TESCAN MIRA LMS, Brno, Czech Republic) was used to analyze the morphology and microstructure of the samples. The structure and composition of the samples were analyzed by X-ray diffraction (XRD) (Bruker D8 Advance, Karlsruhe, Germany). Light absorption was measured by UV-Vis-NIR spectrophotometer (Shimadzu UV-3600i plus, Tokyo, Japan). The water contact angle of the material surface was analyzed by contact-angle measurement (Data physics OCA25, Stuttgart, Germany).

### 2.4. SSG Experiment

**Figure 2:**
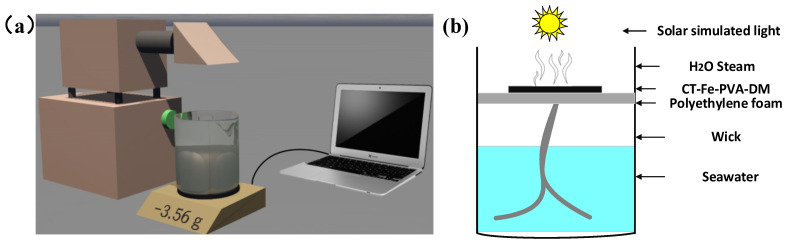
(**a**) Schematic diagram of the laboratory water evaporation measurement system. (**b**) Structure diagram of the evaporator.

The platform schematic diagram of the SSG experimental device is shown in Figure 2a. The photothermal conversion sample was placed in a beaker containing sewage/seawater, the beaker was placed on an electronic balance connected to a computer, a xenon lamp was used to simulate the sun irradiate of the sample and then water evaporation occurred. The mass change of water in the beaker was monitored and recorded in real time. A schematic diagram of the photothermal film evaporator is shown in Figure 2b; the prepared sample was used as the photothermal conversion material at the top, photothermal conversion was carried out on the surface of the sample and the moisture in the interface area was evaporated. The sample to be tested was placed on a piece of polyethylene foam and the polyethylene foam was suspended and fixed in the beaker. On the one hand, it blocked the direct contact between sunlight and water in the beaker, forming a thermal radiation-effect experiment; on the other hand, it reduced the thermal convection between the top photothermal film and the lower seawater. Several cotton wicks were used to pass through the foam, and the two ends were in contact with the sample and seawater. A xenon lamp (CEL-HXF300-T3, CEAULIGHT, Beijing, China) was used to simulate the sunlight and an electronic balance (JS-A5, CEAULIGHT, Suzhou, China) connection with a computer was used to measure the mass change of water. An infrared radiation imager (PTi120, Fluke, Shanghai, China) was used to measure the surface temperature and take infrared radiation images, and an optical power meter (CEL-FZ-A, CEAULIGHT, Beijing, China) was used to calibrate the intensity of simulated sunlight.

### 2.5. SSG Test 

The SSG test was carried out under 1-, 2- and 3-sun irradiation. A light irradiation intensity of 1~3 kw m^−2^ was achieved by adjusting the power of the solar simulator; the upper surface area of the samples used in the experiment was 4 cm^2^; all experiments were completed under the condition that the ambient temperature was 25 °C and the relative humidity was 60%. The mass fraction of simulated seawater used in the experiment was 3.5 wt% and the volume was 150 mL; after being placed for 0.5 h, the experiment began after the seawater soaked the whole sample, and the changes in seawater weight and sample temperature were monitored by an electronic balance and infrared radiation imager, respectively.

The evaporation mass of water can be measured by the balance, and the evaporation rate and evaporation efficiency of water can be obtained by further calculation. The calculation formula of the water evaporation rate (*v*) (kg m^−2^ h^−1^) is as follows [28]:

(1) $$v=\frac{m}{\Delta t\times A}$$

In the above formula, *m* refers to the difference between the water quality lost under simulated light and that without light; Δt is the evaporation time; and A refers to the illumination area of the sample, which is 4 cm^2^ in this experiment.

The evaporation efficiency (η) of water represents the photothermal conversion efficiency of the sample, which can be calculated using the formula shown below [28]:

(2) $$\eta =\frac{v\times h_{v}}{P_{0}}$$

In the above formula, η is the evaporation efficiency of water, *v* is the evaporation speed of water, $h_{v}$ is the total evaporation enthalpy change of water (2260 kJ kg^−1^ [28]) and *P*_0_ refers to the simulated solar radiation input power (1 kW m^−2^).

### 2.6. Solar Water Treatment

The concentration of Na^+^, Mg^2+^, K^+^ and Ca^2+^ in seawater before and after desalination and the concentration of Cu^2+^, Ni^2+^, Cd^2+^, Zn^2+^ and Co^2+^ in sewage before and after purification were measured by inductively coupled plasma (Agilent 5110, Santa Clara, CA, USA). In the experiment, sea salt was used to prepare simulated seawater, and organic dyes (10 mg L^−1^ MO and 10 mg L^−1^ MB) were used to prepare simulated wastewater; the purification performance of organic dyes was tested by UV-Vis-NIR spectrophotometer (Shimadzu UV-3600i plus, Kyoto, Japan).

## 3. Results and Discussion

### 3.1. Characterization of Samples

**Figure 3:**
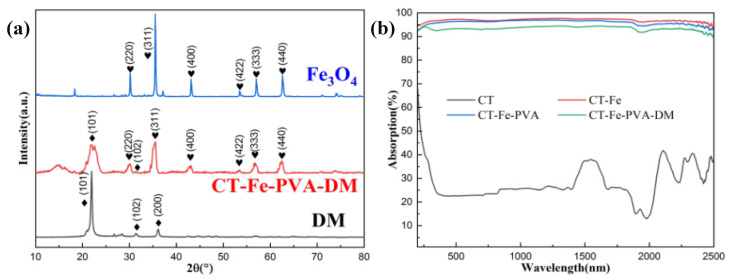
(**a**) XRD diffraction patterns of Fe_3_O_4_, DM and CT-Fe-PVA-DM. (**b**) Light absorption spectra of CT, CT-Fe, CT-Fe-PVA and CT-Fe-PVA-DM.

The XRD spectra of Fe_3_O_4_, diatomite and CT-Fe-PVA-DM are shown in Figure 3a. The diffraction peaks of Fe_3_O_4_ mainly appear at 30.08°, 35.43°, 43.06°, 53.42°, 56.94° and 62.53°, which are located in the (220), (311), (400), (422), (333) and (440) planes, respectively. Diatomite mainly has (101) and (102) characteristic peaks at 21.95° and 31.37°. For the composite sample CT-Fe-PVA-DM, the above characteristic peaks appear, but the intensity of these characteristic peaks is weakened to varying degrees, which may be caused by the alternating arrangement of particles after the mixing of Fe_3_O_4_ nanopowder and diatomite particles. There are some unspecified characteristic peaks in the XRD spectrum of the sample CT-Fe-PVA-DM, which may be the characteristic peaks of cotton and PVA. One of the decisive factors to improve the efficiency of SSG is the light absorption rate of the sample; the light absorption rates of CT, CT-Fe, CT-Fe-PVA and CT-Fe-PVA-DM were measured by UV-Vis-NIR spectrometer (Figure 3b), The average absorption of the four samples was 28.03%, 96.13%, 95.93% and 93.42%, respectively. It can be found that the absorption of the samples added with Fe_3_O_4_ improved significantly, and there is a high absorption in the wavelength range of 200~2500 nm. Moreover, the absorptions of CT-Fe and CT-Fe-PVA are closed, while the absorption of CT-Fe-PVA-DM shows a slight low. Although the surface of the sample becomes rough and the reflectivity of the sample decreases, the absorption of diatomite exhibits as low, and the overall absorption rate of the sample decreases [29].

**Figure 4:**
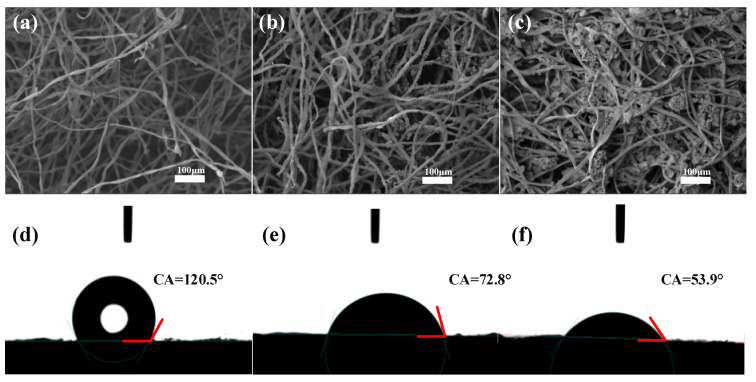
SEM images of the (**a**) CT, (**b**) CT-Fe and (**c**) CT-Fe-PVA-DM; contact angles of (**d**) CT-Fe, (**e**) CT-Fe-PVA and (**f**) CT-Fe-PVA-DM.

The morphology and microstructure of CT; CT-Fe and CT-Fe-PVA-DM were observed by SEM. As shown in Figure 4a–c, the surface of the CT sample is relatively smooth and fluffy, and attached nanoparticles can be seen on the surface of the CT-Fe sample, which looks denser than CT. The CT-Fe-PVA-DM sample looks denser; many attached nanoparticles can also be seen on the surface, and some nanoparticles appear to agglomerate due to the existence of PVA, which makes the particles in the sample glue together to form a block, and the sample becomes solid. The contact angles of the CT-Fe, CT-Fe-PVA and CT-Fe-PVA-DM samples are shown in Figure 4d–f. Compared with the fluffy CT-Fe samples, the contact angle is 120.5°; due to the addition of PVA, the CT-Fe-PVA samples become dense and show better hydrophilicity, with a contact angle of 72.8°; the sample CT-Fe-PVA-DM contains diatomite, and studies have shown that the surface of diatomite contains many hydroxyl groups [30], which increase the hydrophilicity of the samples, and the measured contact angle is 53.9°.

**Figure 5:**
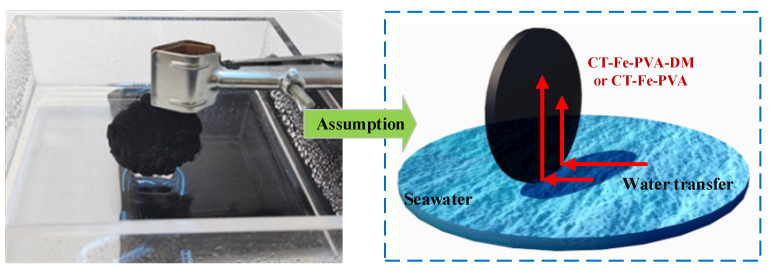
Hydrophilic experimental design of CT-Fe-PVA-DM and CT-Fe-PVA.

**Figure 6:**
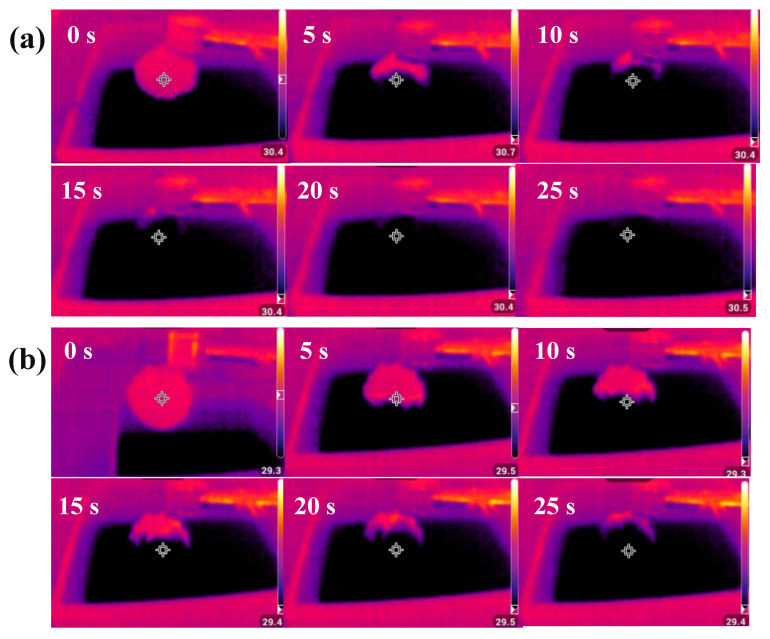
(**a**) CT-Fe-PVA-DM and (**b**) CT-Fe-PVA infrared radiation images corresponding to different times after contact with water.

To compare the hydrophilicity of CT-Fe-PVA and CT-Fe-PVA-DM, the two samples were suspended in a small pool (Figure 5). If the material is hydrophilic, the water must fill the whole sample due to capillarity. The infrared radiation images of the CT-Fe-PVA and CT-Fe-PVA-DM samples at different times after contact with water are shown in Figure 6a,b. The material temperature of CT-Fe-PVA-DM was higher before contact with water, and the infrared radiation image of the sample surface was red. After contact with water, the water quickly began to spread along the sample, and the temperature of the sample surface rapidly decreased. After 5 s, it can be seen from the infrared radiation image that the water had covered half of the sample, and after 15 s, the water had covered the whole sample. The infrared radiation image of CT-Fe-PVA before contact with water is similar to that of CT-Fe-PVA-DM. After contact with water, the moisture also spread along the sample; after 5 s, the moisture only spread to 1/3 of the sample, and it covered the whole sample after approximately 30 s. The results show that CT-Fe-PVA-DM has strong hydrophilicity and water absorption [31], which is a very important performance for the efficiency of SSG, and the infrared radiation image of CT-Fe-PVA-DM is clearer than that of CT-Fe-PVA, indicating that the water distribution is more uniform. These results prove that DM can increase the water absorption and hydrophilicity of the samples.

In the process of sample preparation, using PVA as the “glue” enhances the adhesion between cotton, Fe_3_O_4_ and diatomite. If PVA is not used, powdered Fe_3_O_4_ and diatomite easily fall off the surface of cotton. Therefore, using PVA as the precursor of photothermal material is conducive to the uniformity and stability of the material. It has also been reported that PVA can prevent Fe_3_O_4_ particles from penetrating and blocking the water delivery channel [26]; by adjusting the mass ratio of PVA to Fe_3_O_4_, the hydrophilicity of the sample surface can be adjusted.

**Figure 7:**
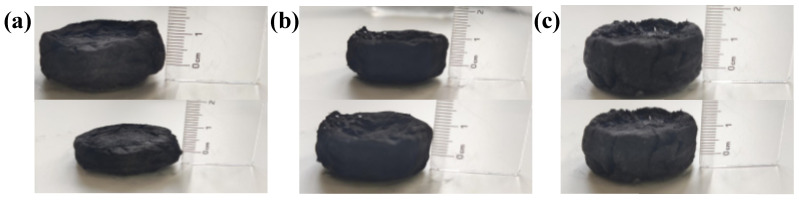
(**a**) CT-Fe, (**b**) CT-Fe-PVA and (**c**) CT-Fe-PVA-DM are aligned before and after extrusion at 1000 g.

The tension and pressure that the sample can bear are also important characteristics of the sample. In reality, many samples cannot be used normally due to tearing, so it is also necessary for the samples to have tension and compression resistance. The force required for the tearing of the sample under the action of an external force was observed using a digital dynamometer (AIGU, Hong Kong). The measured results are shown in Table 1. CT and CT-Fe need only approximately 1.5 N to tear, while CT-Fe-PVA and CT-Fe-PVA-DM need 12 N and 11 N, respectively. The hardness of the sample increases by 8 times after adding PVA, which indicates that PVA can increase the tensile strength of the sample. It can also be seen from Figure 7a–c that CT-Fe has a huge deformation under the weight of 1000 g, while CT-Fe-PVA and CT-Fe-PVA-DM have almost no change, mainly due to the addition of PVA in the latter two samples, which forms a dense three-dimensional structure, and the hardness increases, which can evenly disperse the applied pressure and increase the compression resistance and tension of the samples [32].

### 3.2. Photothermal Conversion and SSG Experiment

**Figure 8:**
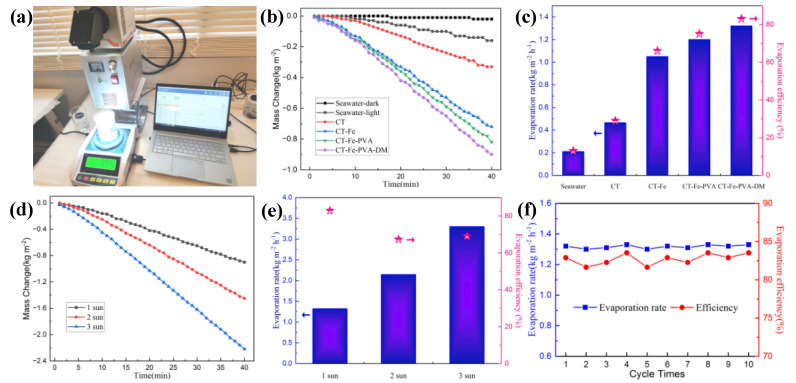
(**a**) Physical picture of the laboratory SSG device. (**b**) The mass change of seawater with illumination time under 1-sun irradiation. (**c**) Evaporation rate and evaporation efficiency of CT, CT-Fe, CT-Fe-PVA and CT-Fe-PVA-DM samples under 1-sun irradiation. (**d**) The mass change of seawater with illumination time under 1-, 2- and 3-sun irradiation. (**e**) Evaporation rate and evaporation efficiency of CT-Fe-PVA-DM under 1-, 2- and 3-sun irradiation. (**f**) Cycle test of CT-Fe-PVA-DM under 1-sun irradiation.

SSG experiments were carried out on pure seawater, CT, CT-Fe, CT-Fe-PVA and CT-Fe-PVA-DM samples using the device shown in Figure 8a. The evaporation performance of several samples was tested, analyzed and compared. As shown in Figure 8b, the evaporation rate of pure seawater is very slow, and its mass changes in the dark and after one-sun irradiation are 0.02 kg m^−2^ and 0.16 kg m^−2^, respectively. In contrast, the evaporation efficiency of the experimental device after using the sample improved to varying degrees; the mass changes of CT, CT-Fe, CT-Fe-PVA and CT-Fe-PVA-DM under one-sun irradiation for 40 min increase successively, which are 0.33 kg m^−2^, 0.72 kg m^−2^, 0.82 kg m^−2^ and 0.9 kg m^−2^, respectively. Further, according to Formula (1), minus the evaporation rate of seawater in the dark (Figure 8b), the evaporation rate order of several samples can be calculated as CT-Fe-PVA-DM (1.32 kg m^−2^ h^−1^) > CT-Fe-PVA (1.2 kg m^−2^ h^−1^) > CT-Fe (1.05 kg m^−2^ h^−1^) > CT (0.47 kg m^−2^ h^−1^) > Seawater-light (0.21 kg m^−2^ h^−1^) > Seawater-dark (0.03 kg m^−2^ h^−1^) (Figure 8c); through Formula (2), it can be further calculated that the evaporation efficiency of seawater, CT, CT-Fe, CT-Fe-PVA and CT-Fe-PVA-DM are approximately 13%, 29%, 66%, 75% and 82.9%, respectively (Figure 8c). This work is superior to most reported data under the same radiation intensity [8,26,27,31,33,34].

Figure 8d shows the curve of the seawater evaporation mass of the sample CT-Fe-PVA-DM with time under one-, two- and three-sun irradiation, The seawater mass change of the sample after 40 min under one-, two- and three-sun irradiation is 0.9 kg m^−2^, 1.45 kg m^−2^ and 2.22 kg m^−2^, respectively; further, it can be calculated that the corresponding evaporation rates are 1.32 kg m^−2^ h^−1^, 2.15 kg m^−2^ h^−1^ and 3.3 kg m^−2^ h^−1^, respectively. From this result, we can obtain that the evaporation rate is not linear with the light intensity, mainly because with the increase in the light intensity, the temperature of the sample surface increases, making the temperature difference between the sample surface and the environment larger, thereby increasing the thermal convection and radiation and reducing the energy utilization. Therefore, the evaporation efficiency decreased from 82.9% of one-sun irradiation to 67.4% of two-sun irradiation and 69% of three-sun irradiation (Figure 8e). Considering the improvement in the evaporation efficiency, the heat preservation capacity of the sample can be increased appropriately on the premise of increasing the light intensity. To evaluate the stability of the evaporation performance under one-sun irradiation, 10 cycle experiments (Figure 8f) were carried out, and each cycle lasted for 1 h. Figure 8f shows that the evaporation rate and evaporation efficiency were stable, at more than 1.3 kg m^−2^ h^−1^ and more than 82%, respectively.

**Figure 9:**
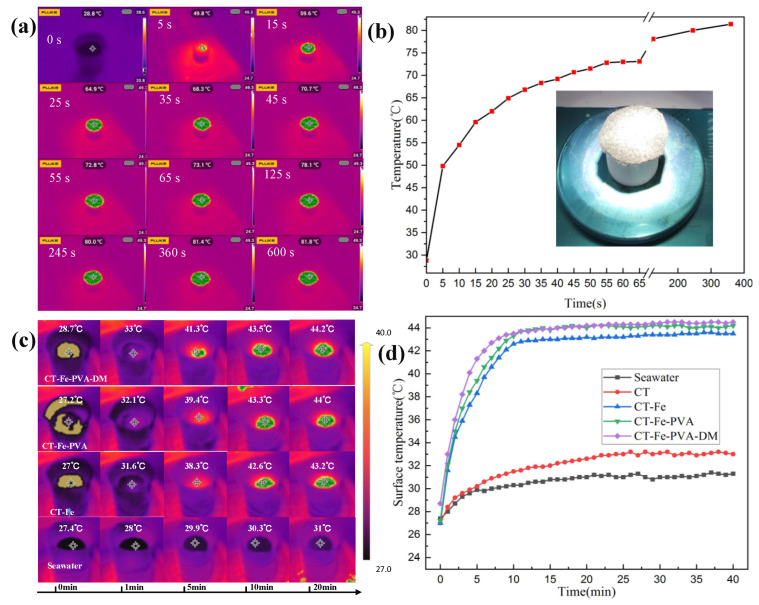
(**a**) Infrared radiation images of CT-Fe-PVA-DM (dry state) at different times under one-sun irradiation. (**b**) Variation diagram of the upper surface temperature of CT-Fe-PVA-DM (dry state) with irradiation time under one-sun irradiation; the illustration shows the real object under one-sun irradiation. (**c**) Infrared radiation images of different samples at different times under one-sun irradiation. (**d**) Variation diagram of the upper surface temperature of different samples with irradiation time under one-sun irradiation.

Figure 9a shows the infrared radiation images of the surface of the CT-Fe-PVA-DM sample at room temperature of 25 °C and relative humidity of 60% at different times under one-sun irradiation. Figure 9b shows the change trend of its surface temperature with time, and the illustration shows the physical picture of the sample when it is irradiated. Within 30 s after irradiation, the surface temperature of the sample increased by 38 °C, and then the temperature rise began to slow; after 65 s, the surface temperature of the sample increased to 73.1 °C; after 6 min irradiation, the surface temperature of the sample finally reached 81.4 °C, and the overall increase was 52.6 °C. The above results show that CT-Fe-PVA-DM as a photothermal material of SSG system has certain practical significance.

Due to the different photothermal conversion efficiencies of different samples, the temperature of the sample surface during the evaporation process is also different. The infrared radiation images of several samples at different times under one-sun radiation are shown in Figure 9c, and the curve of the sample surface temperature changing with time under one-sun radiation is shown in Figure 9d. In the case of pure seawater or CT samples, the temperature rises relatively slowly; when CT-Fe, CT-Fe-PVA and CT-Fe-PVA-DM are used as photothermal conversion films, the temperature rises relatively fast; in the first 5 min, the temperature of the three samples rises by approximately 13 °C, and the temperature of the three samples rises relatively slowly from the 5th minute to the 10th minute, rising by approximately 4 °C. Fe_3_O_4_ is contained in the three samples, indicating that Fe_3_O_4_ has good photothermal conversion ability [35]; after 10 min, the temperature of the surfaces of several samples tends to be stable, in which CT-Fe-PVA-DM is higher than CT-Fe and CT-Fe-PVA, and the surface temperatures are 0.3 °C and 1 °C, respectively. The above data confirm that the samples have good photothermal conversion performance.

The main reasons why the CT-Fe-PVA-DM sample has a higher evaporation rate and evaporation efficiency are as follows: (1) the sample not only has less reflected sunlight, heat conduction loss, heat convection loss and heat radiation loss, but also has higher photothermal conversion efficiency [36]; (2) the sample contains diatomite, which greatly improves the hydrophilicity of the sample so that the bottom water can be quickly and effectively supplied to the surface of the sample, which is conducive to the evaporation of water [37].

### 3.3. Water Treatment Performance

**Figure 10:**
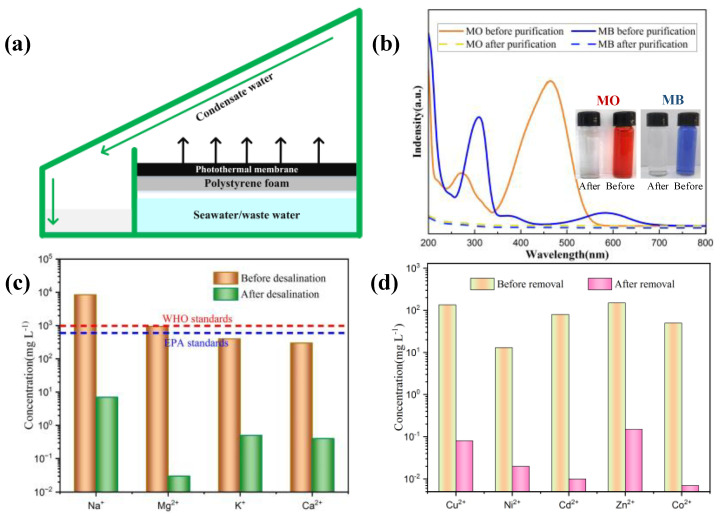
(**a**) Schematic of the lab-built solar-driven evaporative collection system. (**b**) UV−Vis absorbance spectra and optical photos (inset) of the wastewater containing MO and MB before and after evaporation. Concentrations of (**c**) Na^+^, Mg^2+^, K^+^ and Ca^2+^ in the actual seawater sample and the treated water. Content of (**d**) Cu^2+^, Ni^2+^, Cd^2+^, Zn^2+^ and Co^2+^ in the simulated wastewater and the treated water.

Figure 10a is a schematic diagram of the solar-driven water evaporation system built in the laboratory; the CT-Fe-PVA-DM sample described in this paper is placed on the surface of foam as a photothermal conversion layer. In the experiment, one of the main reasons for the high evaporation efficiency of water is that the cotton wick is used as the channel to transmit water, which is also the only path for sunlight to make contact with the water. Polyethylene foam is used as the heat insulation layer to further prevent seawater/sewage from directly coming into contact with sunlight over a large area. The water vapor floats to the inner side of the device and condenses into small droplets, and then falls to the lower end of the device for collection [38,39,40,41].

To further investigate the ability of CT-Fe-PVA-DM to purify wastewater, a solution was prepared with methyl blue (MB) and methyl orange (MO) to simulate wastewater (Figure 10b). The figure shows the absorbance spectra of wastewater and treated fresh water, and the illustration shows the image comparison before and after wastewater purification; it can be seen from the figure that the absorbance of the purified wastewater is close to zero and becomes clear and transparent, indicating that the impurity particles in the wastewater have been removed [33,42,43,44]. As shown in Figure 10d, the comparison diagram of heavy metal ions in the wastewater before and after purification shows that the concentrations of Cu^2+^, Ni^2+^, Cd^2+^, Zn^2+^ and Co^2+^ after purification were reduced by 4, 3, 3, 3 and 4 orders of magnitude, respectively (Table 2), which shows that heavy metal ions have almost disappeared in wastewater; therefore, SSG based on CT-Fe-PVA-DM is an effective method for sewage purification. In addition, we also simulated the experiment of seawater desalination using sea salt to prepare a solution with a mass fraction of 3.5% to simulate seawater; the concentrations of Na^+^, Mg^2+^, K^+^ and Ca^2+^ in the seawater before and after desalination decreased by 3, 4, 3 and 3 orders of magnitude, respectively (Figure 10c, Table 3), from exceeding WHO standards before desalination to below WHO standards after desalination [34,45], meeting people’s drinking water standards. It is fully confirmed that SSG based on CT-Fe-PVA-DM is an effective seawater desalination method.

## 4. Conclusions

Cotton, Fe_3_O_4_, PVA and diatomite were used as raw materials and CT-Fe-PVA-DM samples were prepared in a simple and low-cost way. Fe_3_O_4_ has a strong photothermal conversion ability; the presence of PVA enhances the adhesion between Fe_3_O_4_ and cotton and increases the hardness of the sample and the internal porous structure; the presence of DM enhances the hydrophilicity of the sample. The average light absorption of this sample can reach more than 93%. Additionally, an SSG device was also designed, in which polyethylene foam is used as the heat insulation layer and insulation material, a cotton wick is used as the water transmission medium and the sample is placed on the foam as the light–heat conversion materials. Under one-sun irradiation, the evaporation rate of the sample was 1.32 kg m^−2^ h^−1^, and the evaporation efficiency was 82.9%. We also studied the application of the sample and device in seawater desalination and sewage treatment, the results show that the concentrations of Na^+^, Mg^2+^, K^+^ and Ca^2+^ ions in seawater can be reduced below the WHO standard, and the concentration of heavy metal ions in purified sewage has decreased by 3–4 orders of magnitude, which has fully reached the standard of drinking water. CT-Fe-PVA-DM has the advantages of simple manufacturing and low cost and has high practical significance in seawater desalination and sewage treatment.

## Figures and Tables

**Table 1:** Force required to tear CT, CT-Fe, CT-Fe-PVA and CT-Fe-PVA-DM.

Type	The Force Required to Break the Samples (Newton)
CT	1.4
CT-Fe	1.6
CT-Fe-PVA	12
CT-Fe-PVA-DM	11

**Table 2:** Cu^2+^, Ni^2+^, Cd^2+^, Zn^2+^ and Co^2+^ ion concentration before and after purification.

Ion Type	Cu^2+^	Ni^2+^	Cd^2+^	Zn^2+^	Co^2+^
Ion concentration before desalination (mg/L)	135	13	80	150	50
Ion concentration after desalination (mg/L)	0.08	0.02	0.01	0.15	0.007

**Table 3:** Na^+^, Mg^2+^, K^+^ and Ca^2+^ ion concentration before and after desalination.

Ion Type	Na^+^	Mg^2+^	K^+^	Ca^2+^
Ion concentration before desalination (mg/L)	8520	950	400	310
Ion concentration after desalination (mg/L)	7.21	0.03	0.53	0.41
